# Prevalence and Impact of Anemia on Basic Trainees in the US Air Force

**DOI:** 10.1186/s40798-016-0047-y

**Published:** 2016-05-11

**Authors:** Kathryn E. Myhre, Bryant J. Webber, Thomas L. Cropper, Juste N. Tchandja, Dale M. Ahrendt, Christopher A. Dillon, Roy W. Haas, Samantha L. Guy, Mary T. Pawlak, Susan P. Federinko

**Affiliations:** 1 Joint Base San Antonio, Adolescent and Young Adult Medicine Service, Fort Sam Houston, San Antonio, TX USA; 2 Joint Base San Antonio, Lackland, San Antonio, TX USA; 3 University of Texas School of Public Health, San Antonio, USA

## Abstract

**Background:**

Anemia has been implicated in adverse health outcomes of athletes and military trainees, ranging from overuse injuries to degraded physical and cognitive performance. The purpose of this study was to investigate prevalence of anemia among US Air Force (USAF) basic trainees, to compare physical performance and discharge rates between anemic and non-anemic trainees, and to determine the risks and relative risks of being discharged for anemic versus non-anemic women and men.

**Methods:**

All USAF basic trainees were screened for anemia between July 2013 and January 2014, during an 8-week basic training course at Joint Base San Antonio-Lackland, TX. Age, sex, screening hemoglobin, anthropometric measurements, initial/final physical fitness assessment scores, and discharge data were collected from trainees. Those identified as anemic (hemoglobin <13.5 g/dL for males and <12.0 g/dL for females) received additional labwork, nutritional counseling, and oral iron-replacement, if indicated. Mean percent improvement was calculated for all performance parameters from beginning to end of training. Anemic trainees were compared to non-anemic trainees by *t* test with Welch modification. Results were stratified by sex and anemia severity with post-hoc Bonferroni correction.

**Results:**

Prevalence of anemia was 12.6 % (*N* = 18,827). Respective prevalence of borderline, moderate, and severe anemia was 12.6, 10.9, and 1.9 % for females and 4.8, 3.8, and 0.3 % for males. Mean 1.5-mile run-time, push-up and sit-up counts improved from beginning to end of training for both anemic and non-anemic trainees (*p* < 0.001 both). Non-anemic trainees had slightly greater run-time improvements than borderline and moderate anemics (female: 17.7 vs. 15.2, and 15.1 % improvement, *p* < 0.05 both; male: 14.9 vs. 13.2, and 13.5 % improvement, *p* < 0.05 both). One-way ANOVA demonstrated statistically significant differences between initial and final fitness data for all measures and both genders (*p* < 0.001) with the exception of final sit-up counts for male trainees (*p* = 0.082). Discharge rate for anemic trainees was 9.0 % (20 % for severely anemic trainees) as compared to 5.7 % for non-anemics.

**Conclusions:**

Anemia was prevalent among USAF basic trainees. Identification and treatment of anemia may optimize physical performance and decrease the rate of medical discharge.

## Key points

- Of the 18,827 trainees who entered US Air Force Basic Military Training between 1 July 2013 and 31 January 2014, 12.6 % were anemic.
- Anemic and non-anemic trainees had significant improvements in all components of their Air Force fitness assessment (1.5-mile run, push-ups, and sit-ups) from the beginning to the end of training.
- Anemic female and male trainees had longer initial average run times, fewer push-ups and fewer sit-ups than non-anemic trainees (*p* < 0.001); that statistical difference persisted through the end of training especially for run-times, but less discrepancy was seen for push-up and sit-up values.
- Discharge and delayed graduation were more common among anemic than non-anemic trainees, with risk increasing based on the severity of anemia at the beginning of training.

## Background

Iron-deficiency and iron-deficiency anemia are common conditions among girls and women in the USA, including those who compete in various sports, such as running, skiing, field hockey, crew, basketball, and softball [3, 8]. Given its association with work performance and endurance [7, 10], iron status is particularly important among athletes and others engaged in physically demanding professions, such as the armed forces [5, 7, 25]. In addition, iron-deficiency and iron-deficiency anemia have been associated with numerous adverse health outcomes, including musculoskeletal overuse injuries [16], impaired immune function [22], and psychological sequelae such as diminished cognitive performance [12] and depressed mood [21].

Iron supplementation in these populations may mitigate these conditions and improve performance. Among iron deficient females without anemia, iron supplementation has been shown to improve endurance [20], work capacity [9], and muscle function [2]. McClung and colleagues demonstrated improved vigor and physical performance, specifically running time, among female basic trainees with iron deficiency following iron supplementation [13, 14]. Similarly, iron supplementation of persons with iron-deficiency and iron-deficiency anemia yielded improvement in biochemical laboratory values and reduction of exercise-induced fatigue and increased work capacity [11].

In light of these and other studies, and acknowledging the continued debate regarding screening asymptomatic persons for anemia, Joint Base San Antonio (JBSA)-Lackland staff began screening all incoming US Air Force (USAF) basic trainees for anemia in July 2013. This study aimed to assess the results of this screening program: first, by a cross-sectional analysis to determine anemia prevalence, stratified by sex and severity; and second, by a retrospective cohort analysis to compare physical performance. Additionally, the study sought to determine the risks and relative risks of being discharged for anemic versus non-anemic women and men.

## Methods

### Study Subjects

All USAF basic trainees receive a number of laboratory tests during their in-processing week at JBSA-Lackland, Texas, prior to beginning an 8-week basic training course. Hemoglobin (Hgb) screening was added on 1 July 2013 and observed through January 2014 in order to identify and correct anemia, with the goals of preventing adverse health outcomes and reducing discharge rates. This study, designed to evaluate the impact of the screening program, was deemed exempt by the Institutional Review Boards (IRB) of Wilford Hall Ambulatory Surgical Center at JBSA-Lackland and University of Texas School of Public Health. The study was performed in accordance with the ethical standards of the Declaration of Helsinki. Waiver of written informed consent was granted by the IRBs as the data were collected as part of routine public health surveillance and completely de-identified by the Air Force Trainee Health Surveillance Team prior to analysis.

### Anemia Definition and Classification

Subjects were defined as anemic utilizing the World Health Organization standards [24] for Hgb measured in grams per deciliter (g/dL): less than 12.0 g/dL for women and less than 13.5 g/dL for men. Anemia was further stratified by severity. Anemia for females was defined as borderline if Hgb was between 11.5 and 11.9 g/dL, moderate if 10.0 to 11.4 g/dL and severe if <10.0 g/dL. Anemia for males was defined as borderline if Hgb was between 13.0 and 13.4 g/dL, moderate if 11.5 to 12.9 g/dL and severe if <11.5 g/dL.

### Laboratory Sampling

All blood samples were collected by antecubital venipuncture and processed locally by the Wilford Hall Ambulatory Surgical Center laboratory located at JBSA-Lackland. The blood was analyzed by the Sysmex XN-3000 to determine Hgb and complete blood count. Ferritin was obtained by immunological agglutination, serum iron was obtained using a FerroZine method without deproteinization, and unsaturated iron binding capacity was obtained by direct determination using FerroZine. All three tests were processed using the Roche Cobas c501. Total iron-binding capacity and iron saturation percentages were calculated by the Cobas c501 instrument based on the measured unsaturated iron-binding capacity and serum iron. Results were entered into the Composite Health Care System, from which they were retrieved for analysis.

### Intervention

Subjects identified as having iron deficiency were treated regardless of Hgb level. Males were treated with a single 325 mg tablet of ferrous sulfate (65 mg elemental iron) twice daily. Females were treated with a single 325 mg tablet of ferrous sulfate three times daily with the option of replacing one tablet with a daily multi-vitamin containing 27 mg of elemental iron, which has been offered to all female trainees as part of routine operations since June 2012 [1]. All iron-deficient trainees received group nutritional counseling by a registered dietician, and trainees with severe anemia or with non-iron-deficient anemia were assessed individually by a medical provider.

### Physical Fitness Testing

The Air Force physical fitness assessment was performed at the beginning and end of the 8-week basic training course as an indicator of aerobic and muscular performance. The test consists of height and weight measurements, followed by 1-min of push-ups, 1-min of sit-ups, and a 1.5 mile timed run. Minimum and maximum performance standards are stratified by sex and age. Fitness assessment results and demographic information were retrieved from the Air Force’s Basic Training Management System.

### Statistical Analysis

The mean and standard deviation of demographic and anthropometric measurements, stratified by sex, were calculated. Body mass index was calculated as weight (in pounds) divided by the square of height (in inches), multiplied by 703. Prevalence of anemia and cumulative incidence of discharge, stratified by sex and severity, were calculated. Prevalence was calculated as the number of trainees in each category divided by the total number of trainees, and prevalence ratios were calculated with chi square. Mean percent improvement was calculated for all fitness parameters (run time, push-up count, sit-up count) from beginning to end of training, and anemic trainees were compared to non-anemic trainees by *t* test with Welch modification. Additionally, non-anemic measures and the three classifications of pre- and post- anemic fitness measures were compared to each other by one-way ANOVA, stratified by gender. Percent discharged was calculated as the number discharged divided by the total number of trainees, and the percent graduated late was calculated as the number graduated late divided by the total number of graduates; chi square was used to compare groups. Discharge was defined as having not graduated from basic training by the time of data analysis in summer 2014, whereas late graduation was defined as having graduated after the expected graduation date at the time of entry.

All fitness and operational results were stratified by sex and anemia severity. A *p* value <0.05 was considered statistically significant, and post-hoc Bonferroni correction was applied to adjust the *p* value for multiple comparisons. Statistical analysis was performed using R-Project for Statistical Computing (http://www.R-project.org) [19].

## Results

### Prevalence

A total of 18,827 persons (14,557 males and 4270 females) entered US Air Force Basic Military Training between 1 July 2013 and 31 January 2014, and thus were screened for anemia during their in-processing week. A demographic and anthropometric overview of the population is provided in Table 1. The overall prevalence of anemia was 12.6%: 8.9 % for males and 25.4 % for females, with further categorization by severity (borderline, moderate, severe) given in Table 2.

**Table 1:** Demographic characteristics

	Female (*n* = 4,270)	Male (*n* = 14,557)
Age (years)	20.9 ± 3.2	20.8 ± 3.0
Height (in)	64.1 ± 2.7	69.6 ± 2.9
Weight, initial (lb)	137.1 ± 18.4	164.7 ± 22.1
Weight, final (lb)	138.7 ± 17.2	166.5 ± 20.1
Body mass index, initial	23.4 ± 2.7	23.9 ± 2.7
Body mass index, final	23.7 ± 2.4	24.1 ± 2.4

All values are mean ± standard deviation

**Table 2:** Prevalence of anemia stratified by sex and anemia status

	Female (*N* = 4,270)	Male (*N* = 14,557)	Total (*N* = 18,827)
Non-anemic	3186 (74.6 %)	13263 (91.1 %)	16449 (87.4 %)
Borderline anemia	538 (12.6 %)	702 (4.8 %)	1240 (6.6 %)
Moderate anemia	467 (10.9 %)	551 (3.8 %)	1018 (5.4 %)
Severe anemia	79 (1.9 %)	41 (0.3 %)	120 (0.6 %)

### Physical Performance

All mean physical performance measures (push-up count, sit-up count, and 1.5-mile run time) improved from beginning to end of training for anemic and non-anemic trainees alike (*p* < 0.001 for all). Anemic trainees had longer average run times, fewer push-ups and fewer sit-ups than non-anemic trainees at the beginning of training (*p* < 0.001; Tables 3 and 4) and that statistical difference persisted through the end of training with the exception of final male sit-up values (*p* = 0.082; Table 4). Of note, the difference in run-times between anemics and non-anemics appears to be the most influenced fitness parameter as compared to less operational difference for sit-ups and push-ups (Tables 3 and 4). Non-anemic females and males improved their run times slightly more than their peers with borderline and moderate anemia, but not statistically different from their peers with severe anemia (Tables 3 and 4).

**Table 3:** Mean female physical performance results (sd) stratified by anemia status

Females (*N* = 3,720)						
		No anemia	Anemia			*p* value^
			Borderline	Moderate	Severe	
1.5 mile run^†^	No.	2753	494	413	60	–
	Initial	15.30 (2.02)	15.99 (2.01)	16.44 (2.09)	16.95 (2.23)	<0.001
	Final	12.59 (1.27)	13.56 (1.39)	13.96 (1.48)	13.90 (1.24)	<0.001
	Improvement	17.7 %	15.2 %*	15.1 %*	15.3 %	–
Push-ups^‡^	No.	2706	481	408	60	–
	Initial	17.00 (8.90)	16.18 (8.93)	14.52 (7.67)	14.76 (6.81)	<0.001
	Final	33.88 (10.27)	32.47 (10.10)	31.22 (8.91)	30.95 (8.89)	<0.001
	Improvement	99.3 %	99.3 %	115.0 %	109.7 %	–
Sit-ups^‡^	No.	2749	492	412	60	–
	Initial	29.28 (10.63)	27.80 (10.78)	26.70 (10.40)	27.87 (10.46)	<0.001
	Final	46.95 (8.69)	46.09 (8.70)	45.29 (8.61)	47.00 (8.20)	0.001
	Improvement	60.4 %	65.8 %*	69.6 %*	68.6 %	–

^†^Mean (standard deviation) in minutes

^‡^Mean (standard deviation) in repetitions

^Comparison of four categories based on one-way ANOVA

*Statistically significant difference compared to non-anemic based on paired two-sample *t* test with Welch modification and post-hoc Bonferroni correction

**Table 4:** Mean male physical performance results (sd) stratified by anemia status

Males (*N* = 13,866)						
		No anemia	Anemia			*p* value^
			Borderline	Moderate	Severe	
1.5 mile run^†^	No.	12,698	642	496	30	–
	Initial	12.30 (1.69)	12.79 (1.71)	12.91 (1.78)	13.33 (1.85)	<0.001
	Final	10.46 (0.97)	11.10 (1.11)	11.16 (1.21)	11.23 (1.15)	<0.001
	Improvement	14.9 %	13.2 %*	13.5 %*	15.7 %	–
Push-ups^‡^	No.	12,694	642	496	30	–
	Initial	37.20 (12.59)	35.56 (12.02)	35.25 (12.25)	34.17 (10.24)	<0.001
	Final	54.75 (12.69)	52.78 (12.41)	53.05 (12.55)	53.37 (10.84)	<0.001
	Improvement	47.2 %	48.4 %	50.5 %	56.2 %	–
Sit-ups^‡^	No.	12,696	642	496	30	–
	Initial	38.61 (10.67)	37.18 (10.46)	37.44 (9.72)	39.93 (10.79)	<0.001
	Final	54.98 (8.69)	54.31 (8.49)	54.54 (8.19)	57.17 (9.65)	0.082
	Improvement	42.4 %	46.1 %*	45.7 %	43.2 %	–

^†^Mean (standard deviation) in minutes

^‡^Mean (standard deviation) in repetitions

^Comparison of four categories based on one-way ANOVA

*Statistically significant difference compared to non-anemic based on paired two-sample *t* test with Welch modification and post-hoc Bonferroni correction

Although females and males in all anemia status categories improved their push-up counts, there were no statistical differences between anemic and non-anemic groups by direct *t* test (Tables 3 and 4). Females with borderline and moderate anemia had significantly greater percent improvement for mean sit-ups than female non-anemics (65.8 and 69.6 vs. 60.4 % improvement, respectively, *p* < 0.005; Table 3). Borderline anemic males had greater mean sit-up improvement than male non-anemics (46.1 vs. 42.4 %, *p* = 0.005; Table 4).

### Discharge

The overall discharge rate for the entire cohort was 6.1 %: 5.8 % for males and 7.2 % for females. The discharge rate was 9.0 % for all anemic trainees and 20.0 % for severely anemic trainees, as compared to 5.7 % for those without anemia (Table 5). The risk of discharge and delayed graduation increased with severity of anemia, both overall and when stratified by sex. Trainees who entered with severe anemia were 3.51 times (95 % CI, 2.44–5.05 times) as likely to be discharged as trainees who entered without anemia (Table 5, Fig. 1). Moreover, severely anemic trainees who were not discharged were 3.40 times (95 % CI, 2.16–5.34 times) as likely to graduate late, as compared to their non-anemic peers (Table 6, Fig. 1).

**Table 5:** Incidence and risk of discharge by anemia status, overall and stratified by sex

	Overall		Female		Male	
	*N* (%)	RR (95 % CI)	*N* (%)	RR (95 % CI)	*N* (%)	RR (95 % CI)
Non-anemic	937 (5.7 %)	1.00 [ref]	207 (6.4 %)	1.00 [ref]	730 (5.5 %)	1.00 [ref]
Borderline anemia	91 (7.3 %)	1.29 (1.05–1.59)	37 (6.9 %)	0.99 (0.71–1.39)	54 (7.7 %)	1.42 (1.09–1.85)
Moderate anemia	98 (9.6 %)	1.69 (1.39–2.06)	48 (10.3 %)	1.48 (1.10–1.99)	50 (9.1 %)	1.68 (1.27–2.20)
Severe anemia	24 (20.0 %)	3.51 (2.44–5.05)	16 (20.3 %)	2.91 (1.84–4.60)	8 (19.5 %)	3.60 (1.93–6.73)

**Fig. 1:**
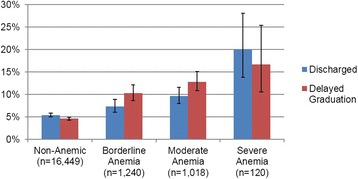
Discharge and Delayed Graduation Rates of Non-Anemic versus Anemic Trainees

**Table 6:** Incidence and risk of delayed graduation by anemia status, overall and stratified by sex

	Overall		Female		Male	
	*N* (%)	RR (95 % CI)	*N* (%)	RR (95 % CI)	*N* (%)	RR (95 % CI)
Non-anemic	761 (4.9 %)	1.00 [ref]	177 (6.4 %)	1.00 [ref]	584 (4.6 %)	1.00 [ref]
Borderline anemia	118 (10.3 %)	2.09 (1.74–2.52)	64 (12.8 %)	2.00 (1.53–2.62)	54 (8.3 %)	1.82 (1.39–2.38)
Moderate anemia	118 (12.8 %)	2.61 (2.18–3.14)	58 (13.8 %)	2.17 (1.64–2.86)	60 (12.0 %)	2.61 (2.04–3.36)
Severe anemia	16 (16.7 %)	3.40 (2.16–5.34)	11 (17.5 %)	2.73 (1.57–4.76)	5 (15.2 %)	3.31 (1.47–7.44)

## Discussion

More than 25 % of females and 8 % of males were found to be anemic upon entrance to US Air Force basic military training. Both the anemic and non-anemic trainees had significant improvements in all components of their Air Force fitness assessment (1.5-mile run, push-ups, and sit-ups) from the beginning to the end of training. The discharge rate for anemic trainees was 9.0 % and for severely anemic trainees was 20.0 %, as compared to 5.7 % for those without anemia.

The high prevalence of anemia in this population is similar to that found in athletes but lower than the general US civilian population [3]. Although not obtained in this study, the prevalence of iron deficiency without anemia appears to be similar among female athletes and trainees and their civilian counterparts [3, 8]. Given increased iron losses via menstruation and lower iron intake than males [3, 12], it is not surprising that female basic trainees had a higher prevalence and greater severity of anemia as compared to male basic trainees.

Given discrepancies in discharge and delayed graduation rates and physical performance parameters between anemic and non-anemic trainees, as found in this study, anemia screening and early treatment may influence retention and physical performance during military training. Screening asymptomatic athletes and warriors for anemia remains controversial. The US Preventive Services Task Force [23] and National Athletic Trainers’ Association [4] do not currently recommend universal anemia screening of asymptomatic adult populations, and no other military services conduct universal screening. The Centers for Disease Control and Prevention, however, has recommended “screen[ing] all nonpregnant women for anemia every 5–10 years throughout their childbearing years,” beginning in adolescence [3]. Such discrepancies may reflect concerns regarding the criteria for an effective screening test [23] and issues with diagnostic accuracy: specifically, low serum iron and Hgb levels in well-trained athletes may either reflect true iron-deficiency anemia [13–15] or a physiologic increase in plasma volume secondary to rigorous physical training, a phenomenon known as “sports anemia” [18, 25]. A corollary argument is that identification and reversal of true anemia at baseline, prior to an intense period of military training, may help prevent exacerbation of anemia.

Additional benefits from screening could be surmised from this study. First, screening allowed for early identification of trainees with critically low Hgb values before commencing an intense exercise regimen. Second, since the majority of anemia cases were attributed to iron-deficiency, low cost treatment with oral iron supplementation was readily available. Third, anemic trainees were discouraged from participating in voluntary blood donation during week 7 of training; in the absence of baseline screening, some trainees with borderline anemia may have donated blood to the detriment of their physical performance. Fourth, three cases of leukemia and lymphoma were diagnosed because of the screening program; it is unclear, however, whether earlier diagnosis had an impact on morbidity or mortality in these cases.

Several drawbacks of screening were also identified. First, although screening tests were inexpensive, diagnostic workups for non-iron-deficient anemia cases were fiscally substantial. Additionally, since the Department of Defense Instruction, DoDI 6130.4 [6] stipulates “anemia that has not been permanently corrected with therapy” is disqualifiable from military service, medical providers were often perplexed by the appropriate military disposition of anemic trainees; this was particularly problematic given the time constraints of basic military training and the inability to demonstrate permanent correction.

Interestingly, improvements in 1.5-mile run time were less dramatic than improvements in sit-up counts among anemic trainees. Overall, anemic female and male trainees had longer average run times, fewer push-ups, and fewer sit-ups than non-anemic trainees at the beginning of training, with persistence of the statistical difference through the end of training, aside from final male sit-up values. However, run-times were more discrepant at the completion of basic training than were push-ups and sit-ups for both genders. This may be related to the aerobic nature of running, which is dependent on efficient oxygen delivery and extraction, or it could be hypothesized that the anaerobic work of strength-based exercises may be more rapidly amenable to iron repletion. It has been noted that iron supplementation in both anemic [7] and non-anemic [11, 17] women with iron deficiency reduces blood lactate accumulation during exertion, which may explain the superior improvements in muscular endurance observed here. In addition to the treatment of anemic trainees, the relatively similar fitness levels between the anemic and non-anemic trainees at the end of training may be explained by the homogenizing effect of a common fitness program or an increased discharge rate among the least fit trainees.

Strengths of this study include a robust sample size, cohort design, access to multiple operationally significant outcomes, and minimal loss to follow-up in a closely tracked military trainee population. The most significant limitation is the lack of a control group (i.e., a group of trainees identified as anemic but not treated), which would determine the efficacy of the iron intervention. Other limitations include lack of iron panel data for all subjects, yielding an unclear picture of the underlying causes of anemia, the possibility of poor trainee adherence to recommended iron replacement regimens, variability of dietary iron intake among anemic and non-anemic trainees, and lack of follow-up Hgb values, especially given the evidence that iron status declines during military training [13, 14].

## Conclusions

In conclusion, this study demonstrates that anemia is prevalent in the military basic training population, specifically among female trainees. The problems of iron-deficiency and anemia and their impact on physical fitness in this population may mirror those among female young adult athlete populations. Further studies are needed to explore the implications of anemia in military and athlete populations and to determine whether the benefits of universal screening outweigh the harms. Future efforts should aim to identify effective and safe countermeasures, including dietary interventions, for the treatment of anemia during military training.

No financial support was received for the conduct of this study or preparation of this manuscript.
